# Adenosine, lidocaine and Mg^2+^ (ALM) fluid therapy attenuates systemic inflammation, platelet dysfunction and coagulopathy after non-compressible truncal hemorrhage

**DOI:** 10.1371/journal.pone.0188144

**Published:** 2017-11-16

**Authors:** Hayley Letson, Geoffrey Dobson

**Affiliations:** Heart, Trauma and Sepsis Research Laboratory, College of Medicine and Dentistry, James Cook University, Townsville, Queensland, Australia; Medical College of Georgia, Augusta, UNITED STATES

## Abstract

**Background:**

Systemic inflammation and coagulopathy are major drivers of injury progression following hemorrhagic trauma. Our aim was to examine the effect of small-volume 3% NaCl adenosine, lidocaine and Mg^2+^ (ALM) bolus and 0.9% NaCl/ALM ‘drip’ on inflammation and coagulation in a rat model of hemorrhagic shock.

**Methods:**

Sprague-Dawley rats (429±4 g) were randomly assigned to: 1) shams, 2) no-treatment, 3) saline-controls, 4) ALM-therapy, and 5) Hextend^®^. Hemorrhage was induced in anesthetized-ventilated animals by liver resection (60% left lateral lobe and 50% medial lobe). After 15 min, a bolus of 3% NaCl ± ALM (0.7 ml/kg) was administered intravenously (Phase 1) followed 60 min later by 4 hour infusion of 0.9% NaCl ± ALM (0.5 ml/kg/hour) with 1-hour monitoring (Phase 2). Plasma cytokines were measured on Magpix^®^ and coagulation using Stago/Rotational Thromboelastometry.

**Results:**

After Phase 1, saline-controls, no-treatment and Hextend^®^ groups showed significant falls in white and red cells, hemoglobin and hematocrit (up to 30%), whereas ALM animals had similar values to shams (9–15% losses). After Phase 2, these deficits in non-ALM groups were accompanied by profound systemic inflammation. In contrast, after Phase 1 ALM-treated animals had undetectable plasma levels of IL-1α and IL-1β, and IL-2, IL-6 and TNF-α were below baseline, and after Phase 2 they were less or similar to shams. Non-ALM groups (except shams) also lost their ability to aggregate platelets, had lower plasma fibrinogen levels, and were hypocoagulable. ALM-treated animals had 50-fold higher ADP-induced platelet aggregation, and 9.3-times higher collagen-induced aggregation compared to saline-controls, and had little or no coagulopathy with significantly higher fibrinogen shifting towards baseline. Hextend^®^ had poor outcomes.

**Conclusions:**

Small-volume ALM bolus/drip mounted a frontline defense against non-compressible traumatic hemorrhage by defending immune cell numbers, suppressing systemic inflammation, improving platelet aggregation and correcting coagulopathy. Saline-controls were equivalent to no-treatment. Possible mechanisms of ALM's immune-bolstering effect are discussed.

## Introduction

Non-compressible torso hemorrhage is the leading cause of potentially preventable death in military and civilian trauma [1–4]. The majority of military deaths occur from hypovolemic-induced cardiovascular collapse in far-forward environments [1], or in civilian settings within 24 hours after injury [5, 6]. Late mortality is often due to activation of an early and excessive systemic inflammatory response, and a triad of complicating factors involving coagulopathy with conspicuous fibrinolysis, acidosis and hypothermia [7–10]. Early traumatic-induced coagulopathy is frequently a bleeding or oozing phenotype, which can evolve over time into a spectrum of clinical states ranging from a worsening systemic hypocoagulopathy [11] to fibrinolysis shutdown [12]. Three key targets of early resuscitation are to: 1) reduce bleeding and fibrinolysis, 2) "rescue and reset" the cardiovascular system, and 3) restore endothelial health. Restoring endothelial health is vital because it is the master controller of inflammation, coagulation, oxygen supply, vascular reactivity and lymphatic function [13, 14]. Currently, there is no fluid that successfully targets these three pillars of resuscitation.

Since 2008 we have been developing a small-volume fluid therapy comprising a bolus of 7.5% or 3% NaCl with adenosine, lidocaine and Mg^2+^ (ALM) bolus for resuscitation and a 0.9% NaCl ALM ‘drip’ for continuum-of-care [15]. The original idea for combining A, L and M came from developing a new ‘polarizing’ cardioplegia, which is in clinical use [16]. At high (0.25 to 1 mM) concentrations, ALM arrests the heart, and at lower ‘non-arrest’ concentrations (10-fold less), it resuscitates the heart. The 'non-arrest' ALM bolus and infusion 'drip' treatments have been shown to protect rat and pig models against regional myocardial ischemia, lethal arrhythmias, cardiac arrest, hemorrhagic shock, endotoxemia, infection and sepsis [15, 17–19]. A, L and M alone do not confer these benefits [15]. In 2016, we showed that the ALM therapy significantly increased survival, improved cardiac function, and reduced internal blood loss by up to 60% in the rat model of hepatic hemorrhage and shock [20]. ALM also corrects early coagulopathy in a number of rat models [11, 15, 18, 21, 22]. The aim of the present study was to examine the effect of small-volume 3% NaCl ALM bolus and 4 hours 0.9% NaCl ALM ‘drip’ on inflammation, hematopoietic-immune and coagulation responses in a rat model of hepatic hemorrhage and shock. We hypothesize ALM therapy will blunt inflammation, decrease immune activation, and correct coagulopathy with preserved platelet function following uncontrolled bleeding in the rat.

## Methods

### Animals and ethics

Male Sprague-Dawley rats (429±4 g) were obtained from James Cook University’s Breeding Colony, Townsville, Australia. All animals were housed in a 14–10 hr light-dark cycle with free access to food and water *ad libitum*, and were anesthetized intraperitoneally with 100 mg/kg sodium thiopentone (Thiobarb; Lyppard, Townsville, Australia). Anesthesia was administered as required throughout the protocol, and depth of anesthesia was monitored via pedal reflex. The study conforms to the Guide for Care and Use of Laboratory Animals (NIH, 8^th^ Edition, 2011) and was approved by James Cook University Animal Ethics Committee (A2118) and US Animal Care and Review Use Office (ACURO). The study was classified as “unconscious without recovery” meaning that any animal death resulting from the different treatments was under anesthesia with no adverse impact on the animal. Animals that survived to end the study (6 hr 15 min after liver resection) were euthanized with Lethabarb (Pentobarbitone sodium, 325 mg/ml).

### Surgical protocol

**Fig 1:**
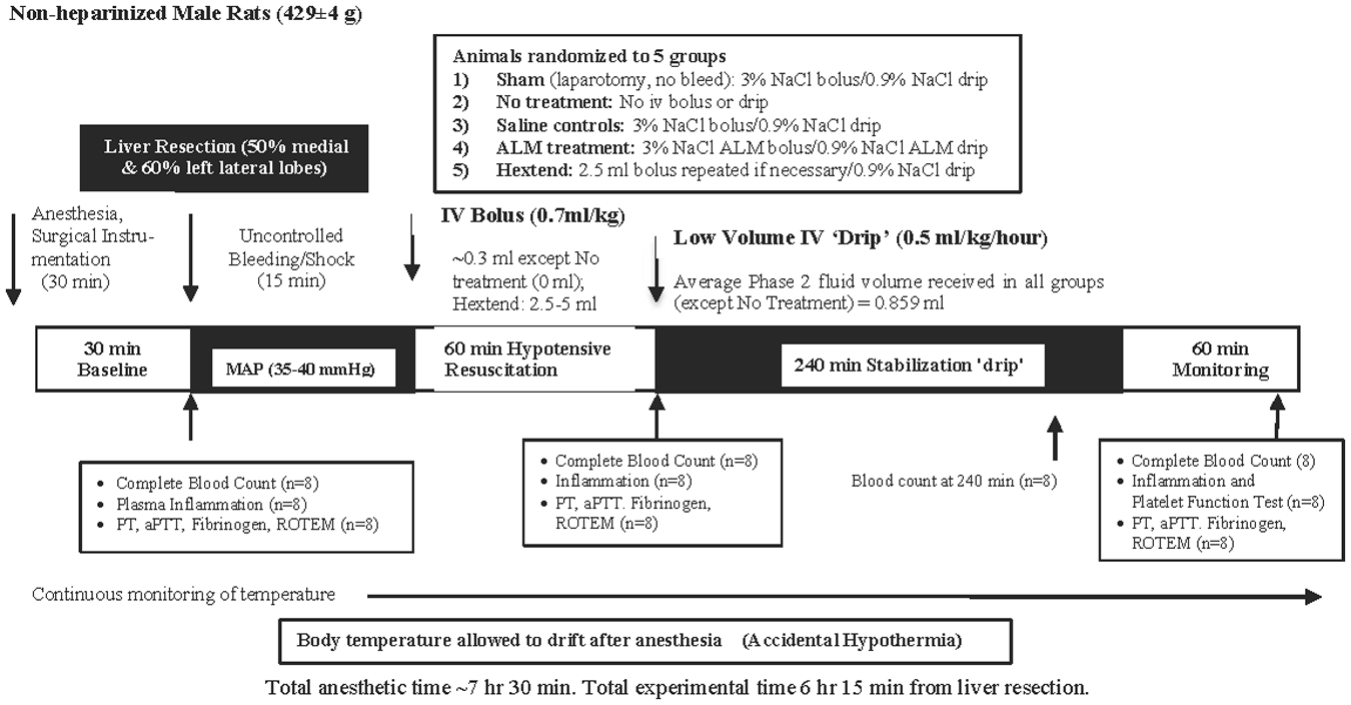
Schematic of the *in vivo* rat model of truncal bleeding and shock treated with two-phase resuscitation therapy. Animals were anesthetized and mechanically ventilated for the entire experimental and monitoring period (~7 hr 15 min). Body temperature was allowed to drift after anesthesia.

The surgical protocol has been described previously by Letson and Dobson [20]. Briefly, following anesthesia, a tracheotomy was performed and animals ventilated on humidified room air. The left femoral vein and artery were cannulated for infusions and hemodynamic monitoring (Powerlab, ADInstruments) [23], and the right femoral artery was cannulated for blood sampling [24]. Following surgical instrumentation, anesthetized animals had a 30-min baseline equilibration period (Fig 1). An additional 8 male Sprague-Dawley rats were anesthetized, ventilated, and had cannulation of the left femoral artery to obtain a 2.5 ml blood sample for baseline measurements of plasma cytokines, ROTEM coagulation analysis, and PT, aPTT and fibrinogen (see below) before humane euthanasia with Lethabarb (Pentobarbitone sodium, 325 mg/ml).

### Uncontrolled bleeding

Uncontrolled hemorrhage was produced via liver injury with excision of 60% of the left lateral lobe and 50% of the medial lobe as previously described [20]. A 2-cm transverse laparotomy exposed the liver for sharp dissection and the cut edges were allowed to bleed freely into the peritoneal cavity. The laparotomy was quickly closed with running suture.

### Experimental design

Fifteen minutes after commencement of hepatic injury, rats were randomly assigned to: 1) Sham (laparotomy without liver resection/bleed, 2) No-Treatment, 3) Saline controls, 4) ALM therapy, or 5) Hextend^®^ (all n = 24) (Fig 1). Any animal that reached MAP<25 mmHg or recovered MAP>90 mmHg prior to Phase 1 resuscitation was excluded from analysis. For Phase 1 resuscitation animals received an intravenous bolus as follows: 1) Sham, 0.7 ml/kg 3% NaCl; 2) No-treatment, no fluid bolus; 3) Saline controls, 0.7 ml/kg 3% NaCl; 4) ALM therapy, 0.7 ml/kg 3% NaCl with 1 mM Adenosine, 3 mM Lidocaine, and 2.5 mM MgSO_4_; 5) Hextend^®^ 2.5 ml bolus (5.94±0.12 ml/kg) + further 2.5 ml if required to maintain MAP>45 mmHg (Fig 1). Seventy-one percent (17/24) of Hextend^®^-treated animals required a second bolus during Phase 1. Sixty minutes after bolus administration, all treatment groups received 4-hr continuous 0.5 ml/kg/hr infusion (Phase 2 resuscitation) as follows: 1) Sham, 0.9% NaCl; 2) No-treatment, no drip; 3) Saline controls, 0.9% NaCl; 4) ALM therapy, 0.9% NaCl ALM (50 mg adenosine, 100 mg lidocaine, 50 mg MgSO_4_/10 ml); 5) Hextend^®^, 0.9% NaCl. Fluids were kept at room temperature (~21°C) and were not warmed prior to infusion. After 4-hr infusion animals were monitored for 60 min (total experimental time from liver injury was 6 hr 15 min) (Fig 1). Consistency of liver injury was ensured by comparing weight of resected liver portions with remaining liver weight. Resected liver weight was 25±0.4% of total liver weight with no significant differences between groups. In order to obtain sufficient blood for measurement of all endpoints, each treatment group had to be repeated three times (n = 24 per treatment) (Fig 1).

### Complete blood counts

The VetScan HM5 Hematology system (REM Systems, Macquarie Park, New South Wales) was used for red cell, hemoglobin, hematocrit, white blood cell, and platelet counts at baseline, 60 min phase 1 resuscitation, and 240 min and 300 min phase 2 resuscitation.

### Plasma cytokine analysis

Milliplex^®^ Rat Cytokine/Chemokine Magnetic Bead Panel (Lot #: 2647883, Abacus ALS, Meadowbrook, Queensland) was used to measure plasma levels of GM-CSF, IL-1α, IL-2, IL-12/p70, IL-4, RANTES, IL-1β, TNFα, IL-6, IL-10, and IFNγ, on Magpix^®^ (Luminex Corporation, Austin, Texas, USA). Assay was carried out according to manufacturer’s instructions. Detection ranges were as follows: GM-CSF, IL-1α, IL-2, IL-12/p70: 4.9–50,000 pg/ml; IL-4, RANTES: 4.9–20,000 pg/ml; IL-1β, TNFα: 2.4–10,000 pg/ml; IL-6: 73.2–300,000 pg/ml; IL-10: 7.3–30,000 pg/ml; IFNγ: 14.6–60,000 pg/ml. Assay sensitivities (minimum detectable concentration, pg/ml), intra-assay precision (%CV), and inter-assay precision (%CV) for each analyte were GM-CSF: 6.8, 2.2, 7.4; IL-1α: 4.2, 2.2, 4.8; IL-2: 5.4, 3.2, 13.3; IL-12/p70: 3.3; 2.2, 7.8; IL-4: 3.1, 3.1, 10.7; RANTES: 1.3, 6.3, 17.1; IL-1β: 2.8, 3.6, 11.3; TNFα: 1.9, 2.7, 10.8; IL-6: 30.7, 2.3, 12.7; IL-10: 2.7, 3.8, 9.0; IFNγ: 6.2, 2.7, 12.4.

### Platelet aggregation tests

Platelet function was assessed in platelet-rich plasma (PRP) using the PAP-8e Platelet Aggregation Profiler and agonists ADP (BioData adenosine-5’-diphosphate reagent, 2 x 10^−4^) and Collagen (BioData soluble calf skin collagen, 1.9 mg/ml). PRP was prepared using a standardized technique of double centrifugation [25].

### Coagulation status

Stago Analysis: Prothrombin time (PT, sec), activated partial thromboplastin time (aPTT, sec), and fibrinogen (g/dL) were measured on the STA Compact (Diagnostica Stago, Doncaster, Australia) using reagents STA Neoplastine Cl (rabbit brain), Triniclot aPTT HS, and STA Liquid Fib. Rotational Thromboelastometry (ROTEM^®^): ROTEM^®^ (Tem International, Munich, Germany) was conducted according to manufacturer’s instructions with all kinetic, elongation and lysis parameters defined in Letson and Dobson [22, 26]. Quality control measurements (ROTROL-N and ROTROL-P) were performed weekly.

### Statistical analysis

*A priori* power analysis to determine sample sizes was conducted using G-power^3^ program to minimize Type 1 errors (MAP 60 min phase 1 resuscitation; n = 8). SPSS Statistical Package 22 was used for all statistical analysis (IBM, St Leonards, New South Wales). All values are expressed as mean ± SEM. Data normality was assessed numerically with Shapiro-Wilks, and Levene’s test was used to determine equality of variances. Parametric data were evaluated using analysis of variance (ANOVA) followed by Tukey’s Honestly Significant Difference or Dunnet’s post-hoc test. Two-way ANOVA comparison was used for within group comparisons. Longitudinal data was analyzed using General Linear Model Repeated Measures ANOVA with Greenhouse-Geisser correction if Mauchy’s Test of Sphericity was not met. Non-parametric data was evaluated using Kruskal-Wallis followed by Dunn’s test. Multiplex cytokine assays were analyzed using MILLIPLEX Analyst 5.1 software (Luminex Corporation, Austin, Texas, USA). Statistical significance was defined as *p*<0.05.

## Results

### Hemoglobin, hematocrit, red cell counts

In shams (no bleed with saline bolus/drip) there was no change in hemoglobin levels but there was a significant fall in red blood cell count (RBC) and hematocrit (HCT) during Phase 1 resuscitation compared to baseline (Table 1). Further falls in red blood cell count and HCT occurred after Phase 2 but these were not significant. In the no-treatment group, hemoglobin, RBC and HCT all significantly fell by 10–14% after Phase 1 and 20–23% after Phase 2. In saline controls, the hematological parameters changed similar to the no-treatment group (Table 1). In contrast, the ALM group had hematological parameters similar to shams after Phase 1 and 2. In both shams and ALM group, RBC, hemoglobin and HCT increased towards baseline after infusion was switched off (see Table 1). Hextend^®^ had significant falls in all parameters over Phase 1 and Phase 2 resuscitation with nearly 30% falls in RBC, hemoglobin and HCT (Table 1).

**Table 1:** Red cell parameters and differential white blood cell counts at baseline, Phase 1 and Phase 2 resuscitation or at the time of death**, and after 60 min monitoring period (end of experiment) in shams, untreated animals, saline controls, ALM therapy group, and Hextend^®^ group.

Group	Resuscitationor Survival Time (min)	HgB (g/L)	HCT (%)	RBC (10^12^/L)	WBC (10^9^/L)	LYM (10^9^/L)	NEU (10^9^/L)	MON (10^9^/L)	LYM (%)	NEU (%)	MON (%)
Sham	Baseline	15.5±0.4	37.7±0.6	7.32±0.09	10.7±1.3	7.6±0.8	2.4±0.4	0.7±0.2	72.8±2.9	21.4±1.7	5.9±1.4
	Phase 1	14.7±0.5	34.7±1.3^¥^	6.76±0.18^¥^	10.9±1.1	4.6±0.7^¥^	5.7±0.4^¥^	0.6±0.1	40.2±3.8^¥^	53.9±4.0^¥^	5.9±0.4
	Phase 2	14.3±1.1	33.7±2.3	6.39±0.46	9.7±2.7	3.6±1.0^¥^	5.1±1.5	1.0±0.3	37.9±3.0^¥^	52.6±2.6^¥^	9.5±1.3^‡^
	Monitoring	15.4±0.9	35.9±1.8	7.06±0.43	9.4±1.7	3.0±0.7^¥^	5.6±1.0^∞^	0.8±0.2	30.6±3.8^‡^	60.8±4.0^¥^	8.6±1.1
No Treatment	Baseline	14.1±0.5	36.6±2.4	7.00±0.46	8.0±1.1	5.8±0.8	1.7±0.3	0.5±0.1	72.2±2.6	20.8±1.6	7.0±1.4
	Phase 1	12.7±0.4*	30.9±1.2^	5.88±0.22*	7.3±0.9*	3.2±0.6^¥^	3.6±0.7^#^^¥^	0.5±0.1	45.3±6.9^¥^	48.4±6.4^¥^	6.3±1.2
	117±13	10.8±1.0*^¥^	29.8±5.3	5.61±1.07	16.6±2.1^‡^	5.3±0.9^§^	9.6±1.3^‡^	1.7±0.2^‡^	32.0±3.4^¥^	57.8±3.6^¥^	10.2±0.6^§^
	Monitoring	(no survivors)									
Saline Control	Baseline	14.3±0.9	35.2±1.1	6.80±0.24	7.9±1.7	6.1±1.2^¥^	1.4±0.5	0.4±0.1	81.8±2.8	14.0±2.6	4.3±0.4
	Phase 1	12.6±0.7*	31.0±1.8^	5.78±0.30*	5.6±0.9*	3.1±0.5	2.3±0.6*	0.3±0.1^#^	56.8±4.7^#^^¥^	38.4±4.5^#^^¥^	4.7±0.4
	298±49	12.0±1.1	28.2±2.5	5.11±0.45^¥^	14.1±2.5^§^	5.4±0.9	7.3±1.5^‡^	1.4±0.3^‡^	40.4±2.7^‡^	49.9±2.8^¥^	9.6±1.1^‡^
	Monitoring (one survivor)	16.5	37.3	7.19	5.30	1.64	3.30	0.35	31.0	62.3	6.7
ALM Therapy	Baseline	15.5±0.4	39.9±0.5	7.47±0.17	11.3±0.9	8.2±0.6	2.3±0.3	0.8±0.1	73.3±2.7	19.8±2.4	7.0±0.9
	Phase 1	14.4±0.3	36.0±1.3	6.79±0.27	10.0±1.2	4.9±0.5^¥^	4.6±0.7^¥^	0.5±0.1	45.0±5.6^¥^	44.5±2.8^¥^	4.6±0.4^¥^
	Phase 2	13.9±0.9	35.1±1.5^¥^	6.63±0.36	8.6±1.9	3.1±0.6^¥^	4.7±1.1	0.7±0.2	38.2±2.1^¥^	52.9±2.2^¥^	8.9±1.3^§^
	Monitoring	15.2±0.8	38.0±1.3	7.44±0.23	10.6±2.3	4.0±1.1	5.4±0.9	1.2±0.3	36.9±2.8^‡^	52.5±4.0^¥^	10.6±1.3^∞^
Hextend^®^	Baseline	14.7±0.8	35.4±1.9	6.74±0.33	8.7±1.0	6.6±0.5	1.6±0.3	0.6±0.2	77.4±2.5	16.7±1.8	5.9±1.1
	Phase 1	12.0±0.6*^¥^	29.3±1.5*^¥^	5.49±0.29*^¥^	5.1±0.5*^¥^	3.7±0.4^¥^	1.2±0.3^†^	0.2±0.0^†^	72.9±3.0^¶^	22.3±3.4^¶^	4.8±0.7
	82±42	10.8±0.5*^‡^	25.8±1.0^‡^	4.84±0.18^‡^	14.4±4.0^§^	6.3±1.5	6.8±2.1^§^	1.4±0.5	49.3±4.6^†^^‡^	41.7±4.1^†^^‡^	9.1±0.8^§^
	Monitoring	(no survivors)									

Data represent mean ± standard error of the mean. HgB = hemoglobin; HCT = hematocrit; RBC = red blood cells; WBC = white blood cells; LYM = lymphocytes; NEU = neutrophils; MON = monocytes. LYM, MON, and NEU are presented as absolute values and percentage of total white blood cell count. n = 8 except as indicated for 60 min monitoring period after Phase 2 resuscitation due to early mortality.

** Mean survival times are from Letson and Dobson (2017).

*p<0.05 compared to Sham and ALM;

^*p*<0.05 compared to ALM;

^#^*p*<0.05 compared to Sham;

^†^*p*<0.05 compared to Sham, No Treatment, and ALM;

^¶^*p*<0.05 compared to all groups;

^‡^*p*<0.05 compared to baseline and Phase 1 resuscitation;

^§^*p*<0.05 compared to Phase 1 resuscitation;

^¥^*p*<0.05 compared to baseline;

^∞^*p*<0.05 compared to Phase 2 resuscitation.

### Differential white blood cell counts

In shams, total white cell count and monocytes were not significantly different throughout the experiment (Table 1). In contrast, lymphocyte counts progressively decreased by 40%, 52% and 61% after Phase 1, 2 and monitoring (*p*<0.05), and neutrophils increased by 2.1 to 2.4 times. These opposing changes in lymphocyte and neutrophil counts were also found when expressed as a percentage of total white cells (Table 1). No-treatment animals experienced a set of complex changes with a significant decrease in total white cell count after Phase 1 (9%) then a twofold increase after Phase 2 (Table 1). The 9% decrease in white cells was associated with a 45% fall in lymphocytes, no change in monocytes, and a 2.1-fold increase in neutrophils. After Phase 2, lymphocyte numbers returned to baseline, monocytes increased 3.4 times (*p*<0.05), and neutrophils increased 5.6 times (*p*<0.05) compared to their respective baseline values (Table 1).

In saline controls, white cell counts underwent similar changes to the no-treatment group. Total white cells significantly decreased at Phase 1 (29%; *p*<0.05) and then increased 1.8 times after Phase 2. Lymphocytes also decreased then rebounded, and monocyte numbers were unchanged after Phase 1 and increased 3.5-times after Phase 2. In contrast to all other cells, neutrophils progressively increased 1.6 to 5.2-fold at Phases 1 and 2 (Table 1). At the end of the experiment, total white cell numbers in saline controls decreased by 33%, lymphocytes decreased by 73%, monocytes decreased by 13%, and neutrophils increased 2.4-fold compared to their respective baseline values. In contrast, ALM-treated animals experienced no change in total white cell counts throughout the experiment, and the differentials changed in magnitude and direction similar to shams (Table 1). Hextend^®^ treatment led to changes similar to no treatment and saline controls.

### Systemic inflammation

In shams, most of the plasma cytokines and chemokines (IL-1α, IL-1β, IL-2, IL-4, IL-6, IL-12, RANTES, GM-CSF and IFN-γ**)** changed little from baseline after Phase 1 (Table 2). Despite TNF-α increasing 2.9-fold and IL-10 increasing 7-fold they were not significantly different from baseline. However, at the end of Phase 2, increases in sham IL-1β (>188-fold), IL-2 (3.3-fold) and IL-6 (109-fold) were significant, and IL-12 was significantly lower (44% lower) relative to baseline (Table 2). Sham IL-1α increased 62-fold and RANTES around 20-fold above baseline but these changes were not significantly different. In contrast to shams, no-treatment had significantly higher IL-1α and β, IL-10 and IFN-γ after Phase 1, and IL-12 significantly decreased by 56%. After Phase 2 only one no-treatment animal survived, and it had high IL-6 (19,278 pg/ml) similar to shams (Table 2).

**Table 2:** Plasma cytokines and inflammatory markers after Phase 1 and Phase 2 resuscitation in shams, untreated animals, saline controls, ALM treatment group, and Hextend^®^ group.

Group	Resuscitation Phase	IL-1α (pg/ml)	IL-1β (pg/ml)	IL-2 (pg/ml)	IL-4 (pg/ml)	IL-6 (pg/ml)	TNF-α (pg/ml)	IL-10 (pg/ml)	IL-12 (pg/ml)	RANTES (pg/ml)	GM-CSF (pg/ml)	IFN-γ (pg/ml)
Baseline		ND	ND	7±3	ND	195±50	14±7	429±269	59±7	607±103	25±2	ND
Sham	Phase 1	2±2	0.1±0.03	4±1	0.2±0.2	195±62	40±11	2940±829	44±11	378±39	35±4	7±3
	Phase 2	124±55	188±70^	23±3^	10±4	21,200±9480^	10±1	377±82	33±7^	1375±351	37±4	44±20
No Treatment	Phase 1	262±104 ^∫^	115±63 ^∫^	71±59	7±4	3732±2137 ^∫^	19±10	1271±372^†^	26±4^	1236±390	38±4	20±5^∞^
	Phase 2 (n = 1)	14	27	34	ND	19,278	22	1753	32	699	30	21
Saline Control	Phase 1	205±132	50±38	8±3^#^	ND	1324±871^#^	46±11	2811±556	35±6	869±308*	35±10	18±11
	Phase 2 (n = 5)	445±84^∫^	618±85^∫^	27±5^∞^	12±10	110,931±21,245^∞^	33±6*	2017±335^∫^	27±3^	1818±312^∫^	43±3	208±100^#^
ALM Therapy	Phase 1	ND	ND	2±1	ND	82±27	11±4	1820±453	24±8	298±86	34±7	3±2
	Phase 2	22±19	32±22^£^	8±2^£^	ND	9142±4000	4±1^£^	197±82	33±7	638±140	35±5	11±3^£^
Hextend^®^	Phase 1	297±65^∫^	166±67^∫^	22±8^#^	11±5*	633±136^∫^	15±9	277±141^‡^	20±2^†^	1099±206*	56±7^¶^	22±7^#^
	Phase 2 (n = 1)	885	830	62	ND	172,100	38	4259	21	3318	42	255

Data represent mean ± standard error of the mean. ND = Not Detected. Baseline values were obtained from 8 healthy anaesthetized, ventilated rats with one femoral cut-down to obtain an arterial blood sample (see Methods). Phase 1 measurements were taken after 60 min bolus resuscitation (75 min after injury). Phase 2 measurements were taken after Phase 1 + Phase 2 resuscitation and one hour monitoring period (375 min after injury). n = 8 unless indicated. No Treatment and Hextend^®^ groups had only 1 survivor at the end of Phase 2 resuscitation, and there were 5 surviving animals in the Saline control group.

^*p*<0.05 compared to Baseline;

^∫^*p*<0.05 compared to Baseline, Sham, and ALM;

*p<0.05 compared to Sham and ALM;

^#^*p*<0.05 compared to ALM;

^∞^*p*<0.05 compared to Baseline and ALM;

^£^ *p*<0.05 compared to Baseline and Sham;

^†^*p*<0.05 compared to Baseline and Saline control;

^‡^*p*<0.05 compared to Saline control and No Treatment;

^¶^*p*<0.05 compared to all groups.

After Phase 1 resuscitation, saline controls showed similar increases in IL-1α, IL-1β, and IL-12 levels as no-treatment. However, IL-10 and TNF-α were twofold higher and IL-6 was 63% lower than the no-treatment group. After Phase 2, IL-1α, IL-1β and IL-4 increased 2.1 times, 12.4 times and 3.4 times respectively compared to their Phase 1 values (Table 2). In addition, RANTES doubled and IFN-γ increased 11.5-fold (18 vs. 208 pg/ml) from Phase 1 to Phase 2. The greatest change in saline-treated rats occurred in IL-6, which increased 85 times from Phase 1 to 2 (1324 vs. 110,931 pg/ml). Interestingly, 3% NaCl ALM bolus led to a cytokine profile similar to baseline after Phase 1. That is, IL-1α, IL-1β, and IL-4 were below the assay's detection limits, and IL-2, IL-6 and TNF-α were less than baseline (Table 2). After Phase 2, all cytokines and chemokines were less than shams. Hextend^®^ treatment at Phase 1 led to similar cytokine levels as no-treatment with the exception of IL-6 and IL-10, which were 83% and 78% less respectively. Other differences were found in RANTES, GM-CSF and IFN-γ, which were 2–3 times higher than no-treatment.

### Coagulation status

#### Phase 1

**Fig 2:**
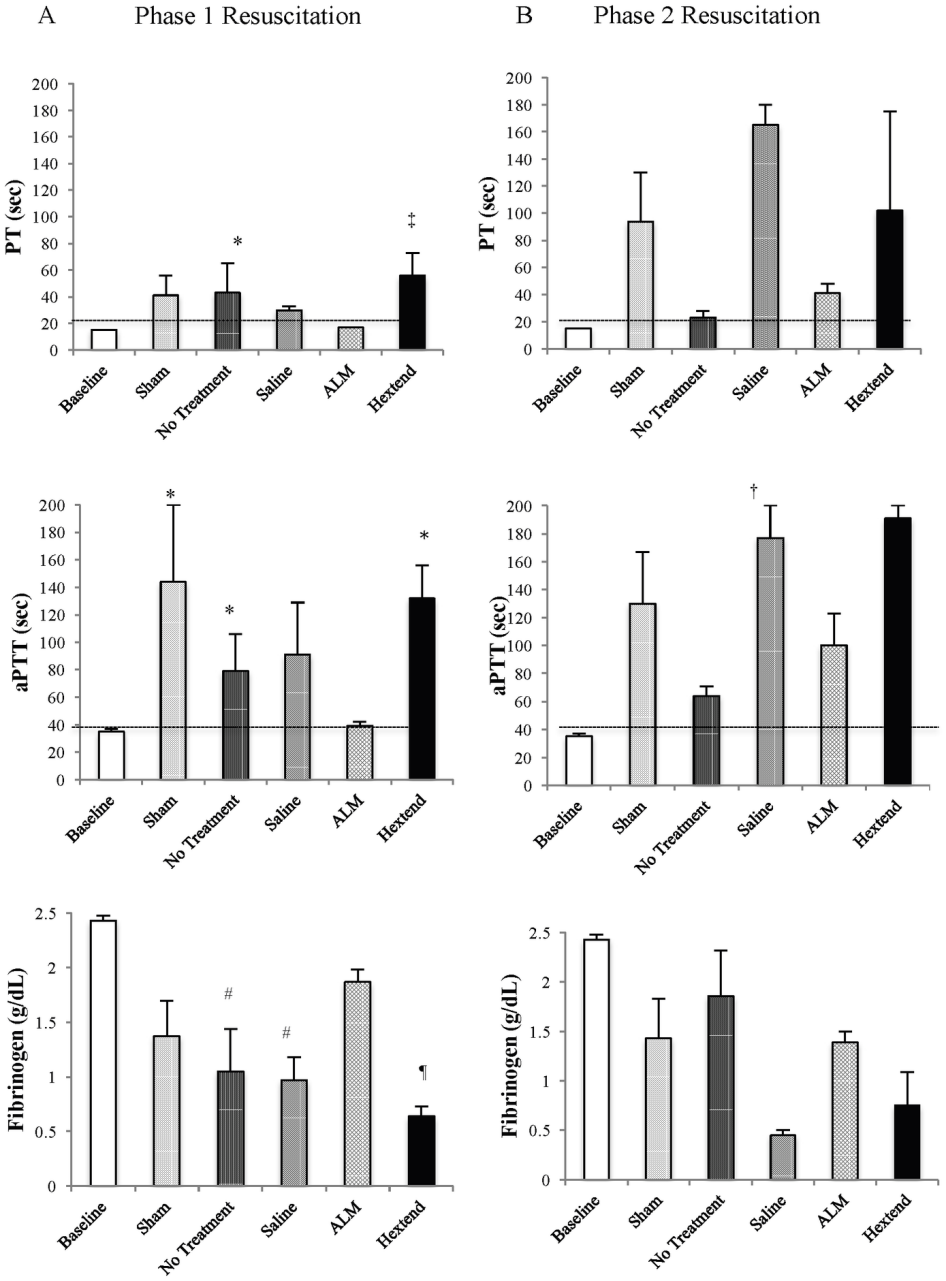
Prothrombin time (sec), activated partial thromboplastin time (sec), and fibrinogen concentration (g/dL) after Phase 1 (A) and Phase 2 (B) resuscitation at baseline, and in shams, no treatment, saline controls, ALM therapy, and Hextend^®^ groups. Values represent mean ± SEM. Baseline values were obtained from 8 healthy anaesthetized, ventilated rats with one femoral cut-down to obtain an arterial blood sample (see Methods). n = 8 for all groups after Phase 1 resuscitation and Baseline, Sham and ALM groups at Phase 2 resuscitation. n = 2 for Hextend^®^ group, n = 3 for No treatment group, and n = 5 for Saline controls at Phase 2 resuscitation due to early mortality. *p<0.05 compared to Baseline and ALM groups; ^#^*p*<0.05 compared to Baseline; ^†^*p*<0.05 compared to ALM group; ^¶^*p*<0.05 compared to Baseline, Sham, and ALM therapy; ^‡^*p*<0.05 compared to all groups except No Treatment group.

Baseline prothrombin time (PT) was 15 sec and increased 2.7-times to 41 and 43 sec for shams and no-treatment after Phase 1 (Fig 2). This increase in PT must be due to the trauma of surgery, as shams did not have the bleed. PT for saline controls, ALM and Hextend^®^ groups were 30, 17 and 56 sec respectively (all n = 8) (Fig 2). These clotting times for all groups were reflected in ROTEM EXTEM and FIBTEM CT with ALM group in EXTEM showing no difference from baseline (46 vs. 40 sec) whereas saline controls were 3.4-times ALM (154 vs. 46 sec) and 3.9-times baseline (Table 3). The prolongation of CT indicates hypocoagulopathy in non-ALM groups (including shams). In saline controls, higher CT was accompanied by slower clot formation times (319 vs. 35 sec ALM), lower alpha angles (58 vs. 83° ALM) and significant reductions in clot amplitude or strength (A10, MCF) (Table 3). Interestingly, there was little difference in lysis index between saline and ALM groups (ML = 3–5%). Similar changes were noted in aPTT and INTEM CT. Baseline aPTT was 35 sec, and increased in shams (144 sec), no-treatment (79 sec), saline (91 sec) and Hextend^®^ (132 sec) groups. ALM aPTT was comparable to baseline (39 sec) (Fig 2). Interestingly, in no-treatment rats there was a significant increase in EXTEM maximum lysis, however, the similar change in APTEM indicates that this was not hyperfibrinolysis. In summary, saline, no-treatment and Hextend^®^ groups were all profoundly hypocoagulable with significant reductions in clot strength but without hyperfibrinolyis.

**Table 3:** ROTEM clot parameters for EXTEM, INTEM, FIBTEM, and APTEM tests after 60 min Phase 1 resuscitation.

Test	Group Phase 1 Resuscitation	Clot Initiation (%)	CT (sec)	α (°)	A10 (mm)	MCF (mm)	LI30 (%)	ML (%)
**EXTEM**	Baseline	100	40±1	83±0.3	70±1	74±1	100±0	6±0.5
	Sham	75	214±155	83±0.7	68±3	60±11	100±0	2±1
	No Treatment	38	162±89	60±21*	43±16^#^	46±15*	88±8	27±16^‡^
	Saline Control	88	154±53	58±10^#^	38±9^#^	44±8*	96±3	5±3
	ALM Therapy	100	46±3	83±0.2	68±1	71±0.5	100±0	3±0.5
	Hextend^®^	63	281±113	57±12^#^	42±9^#^	49±6*	92±5	11±7
**INTEM**	Baseline	100	92±5	85±0.2	73±0.5	76±0.5	100±0	6±1
	Sham	50	462±299^†^	65±14^†^	52±13	67±4	100±0	3±1
	No Treatment	38	458±146^†^	26±18*	21±14*	25±13^#^	99±1	6±6
	Saline Control	75	349±227	46±11*	35±11*	45±10*	100±0	3±2
	ALM Therapy	100	255±73^†^	79±3	66±2	72±1	100±0	2±0.5
	Hextend^®^	38	680±251^†^	45±35^†^	23±17*	27±15^#^	86±15	20±20
**FIBTEM**	Baseline	100	36±1	79±1	15±1	16±1	99±0.5	3±1
	Sham	75	587±525^†^	72±2	12±1	13±1	99±1	3±1
	No Treatment	25	86±30*	59 (n = 1)	10±2	12±2	100±0	0±0
	Saline Control	75	325±162*	75 (n = 1)	6±2	6±2	100±0	1±0.5
	ALM Therapy	100	45±2	75±1	12±1	13±1	98±1	5±1
	Hextend^®^	50	400±218*	68 (n = 1)	7±2	9±2	100±0	0±0
**APTEM**	Baseline	100	43±1	84±0.3	70±0.5	73±0.5	100±0	6±1
	Sham	63	83±23	81±2	66±2	70±1	99±1	6±2
	No Treatment	25	155±58	53±28*	37±25	41±22	79±21	26±26
	Saline Control	75	236±103	63±13*	35±11^#^	39±12	96±4	9±4
	ALM Therapy	100	51±2	83±0.3	67±1	70±1	100±0	5±1
	Hextend^®^	63	388±163*	52±13^#^	39±10*	46±7	87±9	16±12

Data represent mean ± standard error of the mean. Clot initiation represents percentage of animals of n = 8 that initiated 2 mm clot formation. CT = clotting time (sec); CFT = clot formation time (sec); α = alpha angle (°); CFR = clot formation rate (°); A10 = clot amplitude 10 min after clot initiation (mm); MCF = maximum clot firmness (mm); LI30 = lysis index 30 min (%); ML = maximum lysis (%). Baseline values were obtained from 8 healthy anaesthetized, ventilated rats with one femoral cut-down to obtain an arterial blood sample (see Methods).

*p<0.05 compared to Baseline and ALM;

^#^*p*<0.05 compared to Baseline, Sham, and ALM;

^†^*p*<0.05 compared to Baseline;

^‡^*p*<0.05 compared to Baseline, Sham, Saline controls, and ALM.

#### Phase 2

Due to early mortality there was only n = 3 for no-treatment group, n = 5 for saline controls and n = 2 for Hextend^®^ group at Phase 2. PT for shams, no-treatment, saline, ALM and Hextend^®^ groups were 94, 23, 165, 41 and 102 sec respectively, and aPTT values were 130, 64, 177, 100 and 91 sec respectively (Fig 2). In Phase 2, ROTEM confirmed hypocoagulopathy but it was more exaggerated than in Phase 1 since no saline controls had the ability to initiate clot formation in INTEM, FIBTEM or APTEM tests (Table 4). Interestingly, sham animals (no bleed) had a progressive hypocoagulopathy in Phase 2 but differed from phase 1 in that clot initiation and strength were both reduced (Table 4). Shams in Phase 1 showed prolonged clot times but could still form viable clots similar to baseline. A worsening hypocoagulopathy in shams may reflect the combination of accidental hypothermia, anesthesia and long ventilation times (see Discussion). In contrast, ALM-treated animals did better than shams and all other groups after Phase 2 resuscitation, despite having slightly prolonged CT and reduced clot strength.

**Table 4:** ROTEM clot initiation and propagation parameters for EXTEM, INTEM, FIBTEM, and APTEM tests after Phase 2 resuscitation following traumatic hemorrhage and shock.

Test	Group Phase-2 Resuscitation	Clot Initiation (%)	CT (sec)	α (°)	A10 (mm)	MCF (mm)	LI30 (%)	ML (%)
**EXTEM**	Baseline	100	40±1	83±0.3	70±1	74±1	100±0	6±1
	(n = 8)	(8/8)						
	Sham	50	175±13	80±3	46±1	52±1	100±0	0.5±0.5
	(n = 8)	(4/8)	(n = 4)	(n = 3)	(n = 4)	(n = 4)	(n = 4)	(n = 4)
	No-Treatment	50	58±14^†^	82±2	65±4^†^	65±4^†^	76±25	48±44
	(n = 4)	(2/4)	(n = 2)	(n = 2)	(n = 2)	(n = 2)	(n = 2)	(n = 2)
	Saline-Control	17	1519	7	6	17	100	0
	(n = 6)	(1/6)	(n = 1)	(n = 1)	(n = 1)	(n = 1)	(n = 1)	(n = 1)
	ALM-Therapy	100	81±22^†^	78±3	58±3^†^	63±3^†^	87±6	18±9
	(n = 8)	(8/8)						
	Hextend^®^	50	55	82	62	65	100	0
	(n = 2)	(1/2)	(n = 1)	(n = 1)	(n = 1)	(n = 1)	(n = 1)	(n = 1)
**INTEM**	Baseline	100	92±5	85±0.2	73±0.5	76±0.5	100±0	6±1
	(n = 8)	(8/8)						
	Sham	25	102±14	78±7	61±8	67±7	100±0	1±1
	(n = 8)	(2/8)	(n = 2)	(n = 2)	(n = 2)	(n = 2)	(n = 2)	(n = 2)
	No-Treatment	50	113±6	82±2	69±1	72±4	87±13	17±15
	(n = 4)	(2/4)	(n = 2)	(n = 2)	(n = 2)	(n = 2)	(n = 2)	(n = 2)
	Saline-Control	0	NA	NA	NA	NA	NA	NA
	(n = 6)	(0/6)						
	ALM-Therapy	75	164±43	62±10^†^	46±8^†^	57±6^†^	88±10	18±14
	(n = 8)	(6/8)	(n = 6)	(n = 6)	(n = 6)	(n = 6)	(n = 6)	(n = 6)
	Hextend^®^	0	NA	NA	NA	NA	NA	NA
	(n = 2)	(0/2)						
**FIBTEM**	Baseline	100	36±0.6	79±1	15±0.5	16±1	99±0.5	3±1
	(n = 8)	(8/8)						
	Sham	38	43±7	77±1	13±1	14±1	100±0	3±3
	(n = 8)	(3/8)	(n = 3)	(n = 3)	(n = 3)	(n = 3)	(n = 3)	(n = 3)
	No-Treatment	50	53±16	71±9	12±1^†^	13±0	100±0	0±0
	(n = 4)	(2/4)	(n = 2)	(n = 2)	(n = 2)	(n = 2)	(n = 2)	(n = 2)
	Saline-Control	0	NA	NA	NA	NA	NA	NA
	(n = 6)	(0/6)						
	ALM-Therapy	100	108±49^†^	70±5	10±1^†^	11±1^†^	99±1	1±1
	(n = 8)	(8/8)						
	Hextend^®^	50	55	76	12	13	100	0
	(n = 2)	(1/2)	(n = 1)	(n = 1)	(n = 1)	(n = 1)	(n = 1)	(n = 1)
**APTEM**	Baseline	100	43±1	84±0.3	70±0.5	73±0.5	100±0	6±1
	(n = 8)	(8/8)						
	Sham	63	204±126	83±0.3	42±15	44±16	100±0	1±0.2
	(n = 8)	(5/8)	(n = 5)	(n = 3)	(n = 5)	(n = 5)	(n = 5)	(n = 5)
	No-Treatment	50	61±18	83±1	61±77	68±4	76±25	32±29
	(n = 4)	(2/4)	(n = 2)	(n = 2)	(n = 2)	(n = 2)	(n = 2)	(n = 2)
	Saline-Control	0	NA	NA	NA	NA	NA	NA
	(n = 6)	(0/6)						
	ALM-Therapy	100	107±44	77±4	58±4	63±3	80±10	15±7
	(n = 8)	(8/8)						
	Hextend^®^	50	60	83	67	72	100	1
	(n = 2)	(1/2)	(n = 1)	(n = 1)	(n = 1)	(n = 1)	(n = 1)	(n = 1)

Data represent mean ± standard error of the mean. n = 8 except where indicated due to early mortality. NA = Not Applicable. Baseline values were obtained from 8 healthy anaesthetized, ventilated rats with one femoral cut-down to obtain an arterial blood sample (see Methods). Clot initiation represents percentage of animals that initiated 2 mm clot formation. CT = clotting time (sec); CFT = clot formation time (sec); α = alpha angle (°); CFR = clot formation rate (°); A10 = clot amplitude 10 min after clot initiation (mm); MCF = maximum clot firmness (mm); LI30 = lysis index 30 min (%); ML = maximum lysis (%).

^†^*p*<0.05 compared to Baseline.

#### Plasma fibrinogen levels

Baseline plasma fibrinogen level was 2.43 g/dL. After Phase 1, values for shams, no-treatment, saline, ALM and Hextend^®^ groups were 1.37, 1.05, 0.97, 1.87 and 0.64 g/dL respectively, and after Phase 2 were 1.43, 1.86, 0.45, 1.39 and 0.75 g/dL respectively (Fig 2).

#### Platelet aggregation

**Fig 3:**
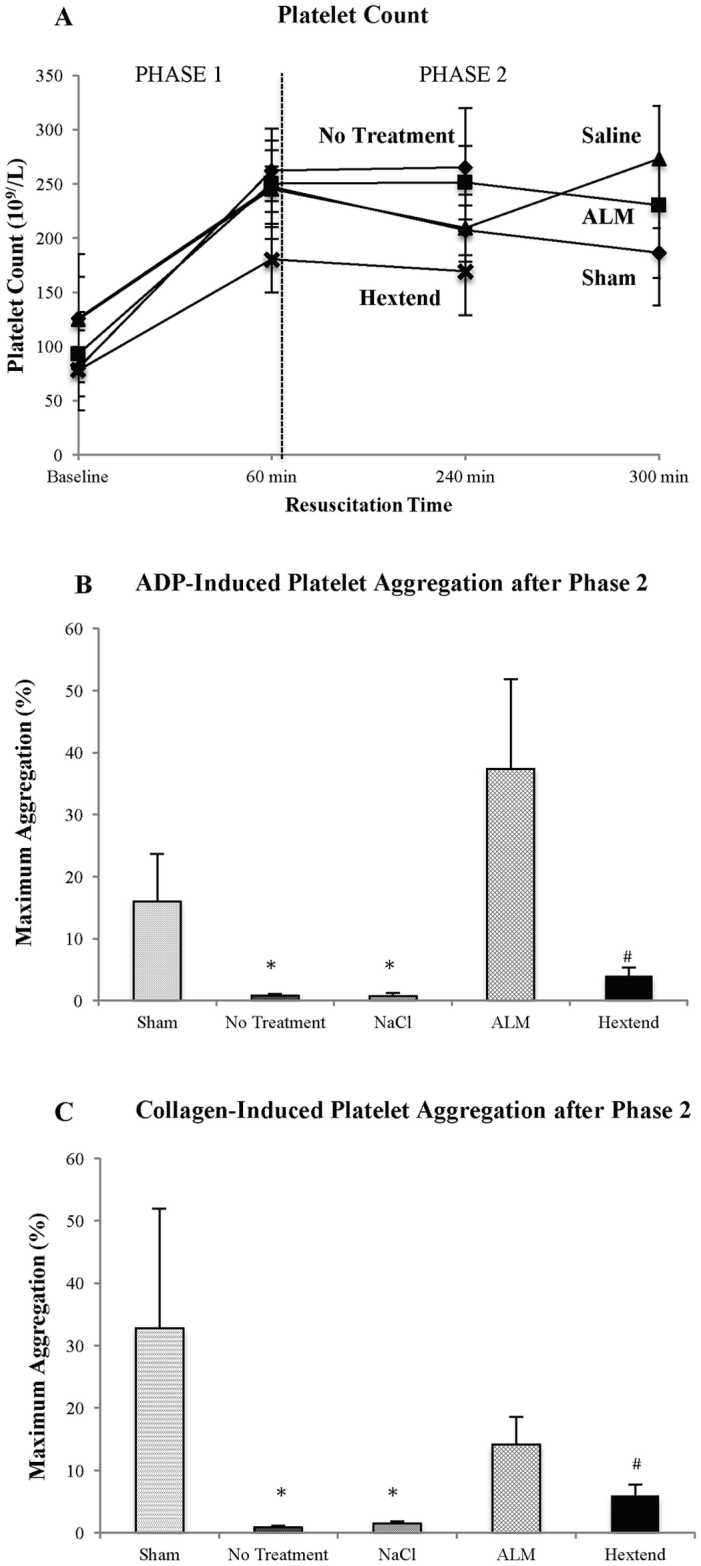
Platelet count (10^9^/L) (A) at baseline, after 60 min Phase 1 resuscitation, and 240 min and 300 min Phase 2 resuscitation, and ADP- (B) and collagen-induced (C) maximum platelet aggregation after Phase 2 resuscitation in shams, no-treatment, saline, ALM, and Hextend^®^ groups. Values represent mean ± SEM. n = 8 for all groups. *p<0.05 compared to Sham and ALM groups; ^#^*p*<0.05 compared to ALM group.

Platelet count in all groups increased by around 2.5 times at Phase 1 then stabilized during Phase 2 or at death (Fig 3A). Maximum ADP-stimulated platelet aggregation for shams, no-treatment, saline controls, ALM and Hextend^®^ were 16%, 0.78%, 0.75%. 37.4% and 4% respectively (Fig 3B). The ALM group ADP-stimulated aggregation was significantly higher than all groups except shams (Fig 3B). Maximum collagen-stimulated platelet aggregation was 33%, 0.89%, 1.5%, 14% and 6% respectively (Fig 3C). ALM group collagen-stimulated aggregation was significantly higher than all groups except shams.

### Rectal temperature

**Fig 4:**
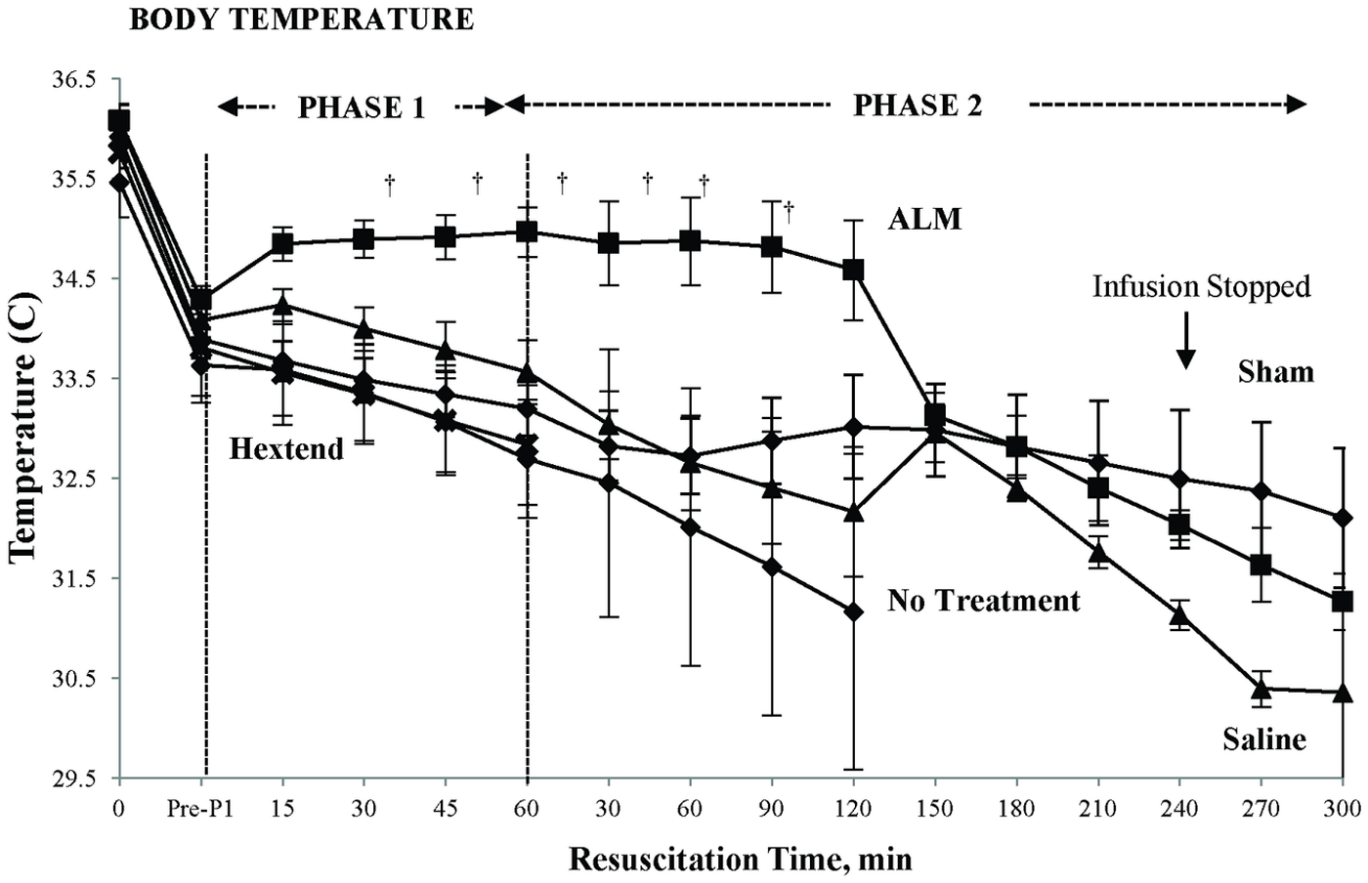
Temperature (°C) at baseline (Pre-bleed), and during Phase 1 and Phase 2 resuscitation in shams, no-treatment animals, saline controls, ALM therapy group, and Hextend^®^-treated animals. Values represent mean ± SEM. All values n = 8 except for groups with mortality. For No-Treatment group n≤7 from 30 min Phase 1; n = 0 from 150 min Phase 2. For Saline controls n≤6 from 45 min Phase 1. For Hextend^®^ group n≤7 from 15 min Phase 1 until 60 min Phase 2; n = 0 from 90 min Phase 2. ^†^*p*<0.05 compared to all other groups.

Temperature in the ALM group was maintained throughout Phase 1 resuscitation and over the first 2 hours of Phase 2 infusion, and then decreased by 1.5°C (~34.6 to 33.1°C) at 150 min, before steadily decreasing to 31.3°C at 300 min (Fig 4). Temperature in saline controls was significantly lower and gradually decreased during Phase 1 from 34.1 to 33.6°C, and this continued in Phase 2 until 150 min when it sharply increased by 1°C, then fell to 30.5°C at 300 min. Sham temperature was 33.8°C before Phase 1 and decreased to 32°C at 300 min (Fig 4).

## Discussion

Systemic inflammation and coagulopathy are major drivers of injury progression and depend upon the type, severity and duration of tissue injury and hemorrhagic shock [1, 2, 12]. We report that small-volume 3% NaCl ALM bolus and 0.9% NaCl ALM 'drip' bolstered the host's defense against non-compressible traumatic bleeding by: 1) defending hematopoietic-immune cell numbers, 2) blunting the systemic inflammatory response, 3) improving platelet function, 4) correcting coagulopathy, and defending body temperature. Saline controls were similar to no-treatment; they were pro-inflammatory, had an activated immune system, lost their ability to aggregate platelets, and were hypocoagulable. Hextend^®^ resuscitation led to the worst outcomes.

### ALM invoked an immune-bolstering defense against trauma and hemorrhage

#### Phase 1 single-bolus resuscitation

Immune effector cells are in high demand following traumatic hemorrhagic shock and can be rapidly taken-up by injured tissues to initiate healing [27, 28]. After Phase 1, we showed that total white blood cells in saline controls significantly fell by 30%, red cell count by 15%, hemoglobin and HCT by 12%, lymphocytes by ~50% and neutrophils increased by ~200% compared to baseline (Table 1). In contrast, ALM-treated animals had total white and red cell counts similar to shams (9–15% losses) (Table 1). A most interesting finding was that the ALM- and sham-groups had nearly identical percentage changes in lymphocytes (40% decrease) and neutrophils (200% increase) as saline controls (Table 1), indicating that this leukopenia-neutrophilia response was largely due to the stress or trauma of surgery (laparotomy, surgical dissection and ventilation), since shams did not undergo liver resection and hemorrhage.

#### Phase 2 ‘drip’ resuscitation

After Phase 2 (or at time of death), red cell counts, hemoglobin and HCT in all non-ALM groups (except shams) continued to decrease (Table 1). However, total white cell numbers nearly doubled and this was associated with a 3-fold increase in monocytes compared to baseline (Table 1). Lymphocyte numbers also rebounded to baseline but neutrophil counts continued to increase in non-ALM groups (Table 1), which may be associated with an end-stage inflammatory response (see below) associated with early mortality [29]. In contrast to Phase 1, these alterations in lymphocyte, neutrophil and monocyte counts were a response to hemorrhagic shock, not the trauma of surgery. A standout result throughout Phase 2 was that the ALM infusion group maintained their differential white cell profile similar to shams (Table 1), implying that ALM mounted an immune-bolstering defense against the 'sterile stressors' of traumatic hemorrhagic shock.

### ALM suppressed systemic inflammation: Possible role of the inflammasome

Interestingly, the reciprocal changes of circulating lymphocyte and neutrophil counts after Phase 1 were not predictive of the degree of systemic inflammation in any group. Saline controls, no-treatment and Hextend^®^ group all developed an increased inflammatory response, which worsened during Phase 2, whereas the ALM-treated animals showed little activation of inflammation with plasma IL-1α and IL-1β levels being below the assay's detection limits, and IL-2, IL-6 and TNF-α below baseline values (Table 2). Similarly, after Phase 2, ALM treatment led to plasma cytokines/chemokine levels that were less than or similar to shams. This suppression effect of ALM during Phase 2 is highlighted by saline controls having up to two orders of magnitude higher IL-1α, IL-1β, IL-2, IL-6, RANTES and IFN-γ levels compared to Phase 1 or baseline (Table 2). Moreover, ALM therapy blunted IL-6 production by ~90% (Table 2), and supported previous findings in a rat model of surgical trauma (single laparotomy) showing reduced IL-6 expression in ALM-treated animals compared with saline controls [30]. The differences in systemic inflammation between ALM and saline-controls in this study most likely reflect differences in the immune cell activation states, not cell numbers, because as stated earlier each group had the same or similar percentage changes in circulating lymphocytes and neutrophils after Phase 1. In conclusion, we showed that the ALM bolus/infusion appears to have provided a permissive environment for healing without inflammation being overexpressed systemically.

While we did not study the underlying mechanisms, ALM therapy may have suppressed systemic inflammation from: 1) blunting receptor recognition of damage-associated molecular patterns (DAMPS) or 'alarmins' that are located on surveillance cells [31–34], and/or 2) suppressing the transition-to-activation of those signaling 'sensors' by blunting the effector responses [35–37]. Endogenous danger signals are largely detected by pattern-recognition receptors (e.g. Toll-like receptors) located on resident and systemic immune cells, including neutrophils, which in turn activate a cytosolic multiprotein complex known as the inflammasome [38, 39]. The inflammasome is an 'effector' signaling platform that releases IL-1, which triggers the induction and amplification of downstream inflammatory cascades to promote healing. However, when IL-1 is overexpressed it can cause excessive inflammation and multiple organ dysfunction [39, 40]. In partial support of ALM inhibition of inflammasome signaling, we showed that IL-1β and IL-1α were undetected in plasma at Phase 1 compared to saline controls (50 and 205 pg/ml respectively), and only increased to ~5% of control values after Phase 2 (Table 2). ALM's ability to blunt systemic inflammation parallels a separate study we recently published in the rat model of polymicrobial sepsis, where we showed that a 0.9% NaCl ALM saline 'drip' administered for 4 hours (total volume 1.6 ml) led to little or no systemic inflammation and 88% survivability after 6 days with no antibiotics [19].

### ALM prevented coagulopathy and defended higher rectal temperatures

In addition to blunting systemic inflammation, ALM therapy corrected coagulopathy, a finding that we have previously reported after pressure-controlled and volume-controlled hemorrhagic shock [11, 22, 23], cardiac arrest [21] and sepsis [15, 41]. In direct contrast, saline-controls, no-treatment and Hextend^®^ animals all showed a worsening of hypocoagulopathy (Fig 2, Tables 3 and 4), which was associated with significantly higher internal blood loss (over 2.5 times) and earlier death compared to the ALM group [29]. ALM correction of coagulopathy was associated with a 1.9- and 3.1-fold higher plasma fibrinogen than controls, and a shift towards baseline values, after Phase 1 and 2 respectively (Fig 2). In the Hextend^®^ group, fibrinogen levels fell by 74% after Phase 1. In contrast to our previous pressure-controlled hemorrhage model (~40% blood loss) with 60 min shock for saline-controls [22, 26], the present model did not trigger hyperfibrinolysis, which may be due to the pressure-controlled model having greater blood loss and prolonged hypoperfusion times leading to more extensive endothelial damage and activation of the protein C and fibrinolytic pathways [11].

During Phase 1, ALM animals also regulated significantly higher body temperatures than the other groups, which may reflect improved hypothalamic thermoregulatory balance from up to 60% less blood loss from early correction of coagulopathy and improved cardiovascular function [29]. After 2 hours into Phase 2, ALM rectal temperature fell by ~1.5°C then slowly decreased to 31.3°C at 5 hours, but interestingly this had no effect on coagulation status. Falls in body temperature in Phase 2 in ALM and other groups may also be associated with differences in blood loss, combined with anesthesia and long ventilation times [42–44].

### ALM therapy improved platelet aggregation function

Differences in coagulopathy among the groups were also mirrored by differences in platelet aggregation measurements. Platelets from ALM-treated animals showed a significant 50-fold higher ADP-stimulated aggregation compared to saline controls and 9.3 times higher in collagen-activated aggregation (Fig 4BC), whereas platelets in saline controls (and Hextend^®^ and no-treatment groups) appeared to have lost their ability to aggregate, which likely contributed to their failure to form a viable clot in ROTEM tests (Fig 4BC). Higher ADP aggregation responses from ALM-treated animals may reflect reduced collagen-linked release of alpha-granules *in vivo* and reduced cytokine production of IL-1β and RANTES, which we found in the ALM group (Table 2). This finding may be clinically significant because Kutcher and colleagues reported in 101 patients that 46% showed decreased platelet aggregation in response to ADP, thrombin receptor-activating peptide, arachidonic acid, and/or collagen, and this defect was associated with a 10-fold increase in mortality [45]. Solomon and colleagues also showed similar results in 163 trauma patients between platelet aggregation defects, reduced clot strength and increased mortality [46].

In addition to improving *in vivo* platelet function, ALM has recently been shown to confer equivalency for *in vitro* storage of cold-stored platelets in platelet additive solution [47]. Interestingly, improvements were shown in the aggregation response to collagen and thrombin receptor activation peptide (TRAP6) by adenosine (A), magnesium (M), A + M and ALM treatment, but not ADP enhancement [47]. In contrast, we found *in vivo* that there was a predominance of ADP enhancement of aggregation, not collagen, which may reflect different mechanisms *in vivo*, and involve a functional endothelium. As with white cell differentials, increases in platelet counts during Phase 1 in all groups, and stabilization in Phase 2 (Fig 3A) were not predictive of differences in the ability to activate and aggregate. Similar increases have been reported in humans following traumatic coagulopathy [48].

### Potential clinical significance

One of the key outcomes of the present study was ALM's small-volume capability (low-cube weight) to blunt immune-inflammatory response to the trauma of surgery and non-compressible blood loss and shock. A total of 0.945 ml was administered over 5 hours for a 350g rat which, if translated, would equate to ~189 ml for a 70-kg human. This may be useful for military medics in far-forward locations or first responders in urban and rural retrieval medicine, who currently do not have many good options for treating non-compressible hemorrhage at the point-of-injury or during evacuation. This may be particularly relevant when blood or blood products are not readily available, or if they are available, may not be the safest option [35, 49–51]. The ALM therapy may also have a role in damage control surgery.

### Limitations of the present study

The present study has a number of strengths and limitations. A possible strength is that the animal model allows the benefits and adverse events of new therapies to be examined in a controlled manner from a 'systems' approach. A major weakness is that whole body studies are exceedingly complex and few animal studies translate into human use. *In vivo* studies are also plagued with the difficulty of unravelling the underlying mechanisms to a particular challenge and require *in vitro* systems. Another limitation is that our animals were anesthetized and positively ventilated for up to 6 hours, which may have affected the physiology of the system, as was reflected in shams. Lastly, we did not examine immune cells in their different activated states during Phases 1 and 2, such as T-cell effector functions and their natural killer subsets, or macrophage M1/M2 phenotypes and neutrophil modulation in response to traumatic hemorrhagic shock.

### Conclusions

Small-volume ALM therapy improved hematopoietic-immune function and attenuated inflammation over 5 hours in the rat model of non-compressible traumatic hemorrhage. Saline-controls were proinflammatory, progressively hypocoagulable and similar to no-treatment. Correction of coagulopathy in ALM-treated animals was associated with a significant ADP- and collagen-stimulated platelet aggregation. Hextend^®^ treatment led to profound inflammation and coagulopathy. ALM therapy appears to have acted like a host-directed immune modulator following hemorrhagic shock and may have applications in the treatment of traumatically injured patients to correct trauma-induced coagulopathy and prevent systemic inflammatory response syndrome and progression to multiple organ failure.
